# A systems approach framework for evaluating tree restoration interventions for social and ecological outcomes in rural tropical landscapes

**DOI:** 10.1098/rstb.2021.0111

**Published:** 2023-01-02

**Authors:** Marion Pfeifer, Susannah M. Sallu, Andrew R. Marshall, Stephen Rushton, Eleanor Moore, Deo D. Shirima, Josephine Smit, Esther Kioko, Lauren Barnes, Catherine Waite, Leander Raes, Laura Braunholtz, Pieter I. Olivier, Evodius Ishengoma, Sam Bowers, Sergio Guerreiro-Milheiras

**Affiliations:** ^1^ Modelling, Evidence and Policy RG, SNES, Newcastle University, Newcastle upon Tyne, NE1 7RU, UK; ^2^ School of Earth and Environment, University of Leeds, Leeds LS2 9JT, UK; ^3^ Department of Environment and Geography, University of York, York YO10 5NG, UK; ^4^ Forest Research Institute, University of the Sunshine Coast, Sunshine Coast, QLD 4556, Australia; ^5^ Department of Ecosystem and Conservation, Sokoine University of Agriculture, PO Box 3010, Morogoro, Tanzania; ^6^ Faculty of Natural Sciences, University of Stirling, Stirling FK9 4LA, UK; ^7^ Southern Tanzania Elephant Program, PO Box 2494, Iringa, Tanzania; ^8^ Entomology, National Museums Kenya, PO Box 40658-00100, Nairobi, Kenya; ^9^ IUCN Centre for Economy and Finance, Washington DC, USA; ^10^ Department of Zoology and Entomology, University of Pretoria, Pretoria 0028, South Africa; ^11^ M.A.P Scientific Services, Pretoria 0145, South Africa; ^12^ College of Science and Engineering, University of Edinburgh, Edinburgh EH8 9YL, UK

**Keywords:** ecosystem services, ecosystem disservices, predictive modelling, systems modelling, forest landscape restoration, coupled human and natural systems

## Abstract

The science guiding design and evaluation of restoration interventions in tropical landscapes is dominated by ecological processes and outcomes and lacks indicators and methods that integrate human wellbeing into the restoration process. We apply a new systems approach framework for tree restoration in forest-agricultural landscapes to show how this shortcoming can be addressed. Demonstrating ‘proof of concept’, we tested statistical models underlying the framework pathways with data collected from a case study in Tanzania. Local community perceptions of nature's values were not affected by levels of self-reported wildlife-induced crop damage. But mapped predictions from the systems approach under a tree restoration scenario suggested differential outcomes for biodiversity indicators and altered spatial patterns of crop damage risk, expected to jeopardize human wellbeing. The predictions map anticipated trade-offs in costs and benefits of restoration scenarios, which we have started to explore with stakeholders to identify restoration opportunities that consider local knowledge, value systems and human wellbeing. We suggest that the framework be applied to other landscapes to identify commonalities and differences in forest landscape restoration outcomes under varying governance and land use systems. This should form a foundation for evidence-based implementation of the global drive for forest landscape restoration, at local scales.

This article is part of the theme issue ‘Understanding forest landscape restoration: reinforcing scientific foundations for the UN Decade on Ecosystem Restoration’.

## 1. Introduction

Forest landscape restoration (FLR) is seen as central to achieving global restoration ambitions to reverse ecological impacts of land degradation and enhance human wellbeing [1]. FLR is meant to bring together diverse disciplines, knowledge and value systems and management practices to restore multi-functional landscapes for multiple benefits to different stakeholders [2]. In theory, this ambition aligns well with the Bonn challenge (see https://www.bonnchallenge.org/) and other targets that are used as a roadmap for global restoration progress, within and beyond the UN Decade on Ecosystem Restoration (2021–2030).

International organizations, countries and private partners have pledged their commitments, advocating for nature-based adaptations to food security, climate change and biodiversity. These may include management of forests to prevent disease spill-over and as safety nets providing food, income, fuel and medicine and restoration of valuable land for climate change mitigation through carbon sequestration (*sensu* ecosystem services). These nature-based solutions may also include the management of crop production landscapes for multi-functionality and resilience to environmental shocks [3], for example through agro-ecology or agroforestry [4].

Millions of people rely on agroforestry farming and recognize the values of trees and native tree species [5–7]. Trees on and around farms regulate ecosystem processes relevant for crops, including water regulation and prevention of soil erosion [8], microclimate buffering [9] and maintenance of populations of pollinator and natural pest control species [10]. Trees can improve soil fertility and health, thereby influencing soil carbon, nutrient cycles and soil biota [11], which are important for crop growth [12]. Yet, forests and trees can also increase risks of human–wildlife conflicts, including crop damage and disease spill-over [13]. Trees may negatively impact yields, yet evidence is limited for tropical humid areas and inconclusive overall, with yield changes due to competition with trees depending on tree species, tree maturity and crop type [14–16].

Using FLR to address interdependent outcomes for ecological and human wellbeing in landscapes used for agriculture is a transformative challenge [17]. These landscapes are socio-ecological systems, in which human and natural components interact in complex and dynamic pathways, shaping food security and biodiversity among other outcomes relevant for sustainable development [3,18]. A systems approach allows us to link elements within and across the human and natural components and to capture the complexity of interactions between them and the system drivers across scales of space, ecology and organization [19]. Applied to FLR, a systems approach could prove essential to predict synergies and trade-offs in outcomes at the food security–biodiversity–climate change nexus [20], resulting from competing demands on land and resources [21]. FLR can enhance ecosystem services and reverse biodiversity loss, but can also have negative consequences for either, if both are treated in isolation [22]. It can bring benefits to people but also real or perceived costs to their wellbeing, through effects on food security [23,24]. Costs can manifest as disservices such as crop damage from wildlife and through reduced access to land [25]. Disservices may, in turn, affect communities' perceptions on the value of trees or other natural features and thus shape decision-making on land management, thereby reshaping biodiversity and food security outcomes. The literature suggests that this relationship differs between community members influenced by, for example, gender, location and livelihoods [26]. Socio-economic conditions, farm size, insecure land tenure agreements and high labour costs, for example, have been shown to be important barriers to tree planting [27,28] and these barriers are experienced differently by men and women [29,30].

Approaches with limited disciplinary diversity have dominated scientific discourse on prioritization of restoration processes and global policy debate. Restoration science has emphasized carbon benefits [31,32] and narrowly defined biodiversity benefits [33,34]. Human wellbeing outcomes, if included in planning or monitoring, remain poorly accounted for, using national census data and top–down understanding that fails to capture the realities of stakeholders, their livelihoods and opportunity costs following restoration [34]. While several wellbeing analysis frameworks have been developed over the past two decades [35,36], postulating ecosystem service values for wellbeing [35,37], progress has been hampered by substantial methodological challenges regarding primary data collection and analyses [21]. Recently, a protocol was introduced for the selection of indicators that can measure local human wellbeing along dimensions of materials, health, social relations, security and freedom of choice [38] and link it to conservation outcomes [39]. Introduced as a bottom–up tool that can be adapted to local contexts, this protocol enables the user to translate the complexity of human wellbeing into locally appropriate measurable indicators and hence can be used as practical and statistically validated step-by-step guidance to design wellbeing assessments.

**Figure 1.**
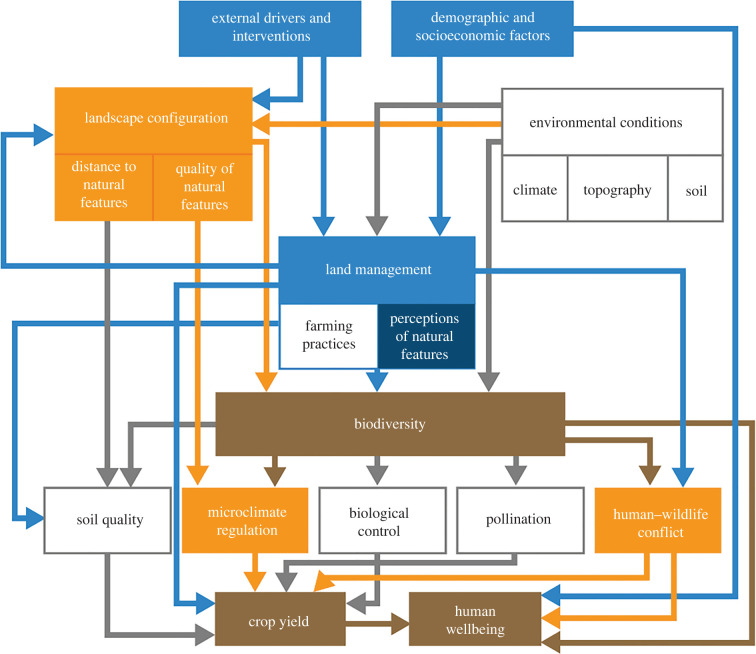
System model underlying the systems approach framework (adapted from [40]). In this study, we focus on three pathways. We assume pathways from landscape configuration, and changes thereof due to land management, to biodiversity (ecological community), microclimate regulation and human–wildlife conflict, expecting relationships with crop yield and wellbeing outcomes in a case study landscape in Tanzania (figure 2). Blue shading represents social components, components in orange originate from the ecological dimension, and brown shading refers to outcomes linked to nature conservation, food security, and human wellbeing. Unshaded boxes represent pathways and drivers in [40] that we did not parameterize in this study evaluating outcomes of a restoration intervention scenario. Governance and adaptive management External drivers and interventions includes governance and decision-making on adaptive management/restoration interventions [41]. For example, incentives used to promote uptakes of restoration interventions [42] and management decisions on what to plant [43] and where to plant [44].

A systems approach framework for crop production landscapes (figure 1) was recently introduced to address method challenges and provide a clear set of indicators and pathways integrating biophysical with social pathways and components in ways that can be measured and modelled [40]. The framework can be calibrated to a specific landscape, and it can then help to develop scenarios that predict the ecological impacts and human wellbeing outcomes of tree restoration interventions before trees are even planted. Governance and adaptive management are included as external drivers and interventions in the systems approach, linking to landscape configuration and land management. This in turn can provide a tool to engage stakeholders [45] for decision-making on 'where to plant’ and ‘what to plant’ and consideration of the development of incentives and strategies to mitigate costs to stakeholders and maximize benefits. The framework thus aligns with calls for participatory restoration [46] and the ‘ten golden rules for forest restoration’ [47], which formally recognize the importance of involving local communities throughout the steps of the restoration process.

**Figure 2.**
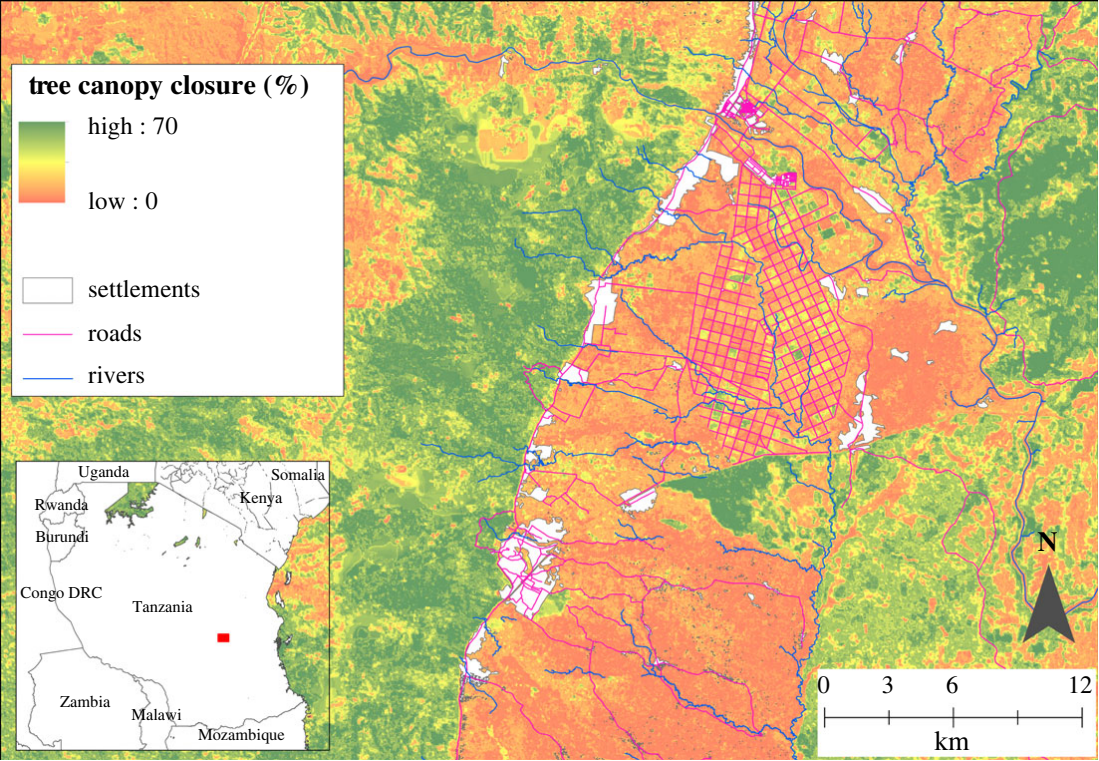
Study landscape of the Agrisys Tanzania research. For details on locations of survey points see S1 in the electronic supplementary material. The restoration scenario (riparian buffer forest restoration) is visualized in green. These are linear structures of 60 m width on either side along riverine areas traversing the valley from east to west, thereby linking wildlife populations in high tree cover habitats and allowing them to move through the valley, which is largely used for crop production.

In this paper, we apply the systems approach framework to one spatially explicit, hypothetical scenario of a FLR intervention in the Kilombero Valley, Tanzania (figure 2). The landscape is part of an agricultural growth corridor, where smallholder farmers sit alongside industrial approaches to farming and are targeted for sustainable intensification of agriculture.

We address three main objectives. First, we aim to demonstrate how framework pathways can be parameterized and used to predict and map synergies, as well as trade-offs both in biodiversity outcomes and in outcomes related to farmer wellbeing following changes in landscape configuration under tree restoration schemes. We model wildlife-induced risk of crop damage expecting a direct negative relationship with wellbeing. Our assumption is that increased crop damage resulting from restoration interventions changing biodiversity outcomes should translate to decreased wellbeing. Crops are locally important and correlated to livelihood and food security of farmers, and 50.4% of households interviewed experienced medium to high levels of food insecurity in 2019. We focus on bird and mammal biodiversity indicators, including threatened species and species providing ecological services (pest control) and disservices (seed- and plant-eating species) to farmers [48]. We model crop damage by elephants, which is one source of human–elephant conflict in the Kilombero Valley, and elsewhere in Africa [49]. Other conflicts, including predator attacks on livestock and people and elephants killing people also impact wellbeing but are not considered here. Second, we draw on social data collected from farmers in the landscape using the new wellbeing measurement protocol and test for a relationship between self-reported human wellbeing and self-reported crop damage caused by wildlife/pests in general or by elephants. We then provide our perspective on how local knowledge and value systems could be incorporated into the planning of the restoration process to increase participation and representation of local communities with planned restoration interventions.

## 2. Material and methods

### (a) Case study landscape and the tree restoration scenario

The study area is located in the north of the Kilombero Valley (figure 1). The landscape is human-dominated and lies nestled between the Udzungwa Mountains National Park (UMNP) to the west and Mikumi and Nyerere National Park (NNP) to the east, which was historically linked through wildlife corridors, most of which have been converted to agriculture in the past two decades [50]. The landscape's land use mosaic comprised forest and savannah ecosystems (protected in forest reserves or managed in village community reserves), settlements and an industrial sugarcane farm, interspersed with smallholder farms—mostly subsistence, less than 2 ha—including monocultures, complex intercropping and a small amount of agroforestry. The main smallholder crops are rice, sugar, maize, okra, cassava and pumpkin. Settlements cluster along the main highway and feeder roads.

The systems approach framework integrates social with ecological factors. It is flexible in that it allows users to select indicators used to measure those components and outcomes depending on local context, thereby allowing users to account for locally relevant adjustments, participative processes or data availability [40]. In this study, we focus on three pathways linking landscape configuration to biodiversity and wellbeing outcomes: (1) landscape configuration (quality of natural features) relationships with microclimate and crop yield, (2) landscape configuration (quality and arrangement of natural features) relationships with biodiversity outcomes (ecological community, traits) and (3) landscape configuration relationships, with risk of elephant-induced crop raiding as an indicator for human wellbeing impacts (figure 1). We model impacts of changes in landscape configuration under the restoration scenario along these pathways.

#### (i) Current landscape configuration

We used remote-sensed and digitized maps as inputs to geographic information systems (GIS) analyses, which created maps of covariates at 30 m resolution that we could use as predictors in our modelling framework. These maps described landscape configurations hypothesized to be important for biodiversity and human–wildlife interactions (see electronic supplementary material, S4 for details). The key variables developed as maps were landcover (five classes: forest, grassland, cropland, water bodies and settlements), canopy closure (% closure per 30 m pixel), distance to forest, distance to river, distance to road, distance to settlements, and distance to industrial plantation, and NDVI (normalized difference vegetation index). Further maps describing the percentage of forests in a 250 m window around each pixel as well as mean and variation (standard deviation) of NDVI and canopy closure in 150 m and 500 m buffers around each pixel were derived using GIS. We also used the UN adjusted population density map for the year 2020 (number people for 1 ha pixels) and the GEDI tree canopy height map (see https://glad.umd.edu/dataset/gedi/).

#### (ii) Landscape configuration under restoration scenario

Our restoration scenario encompassed the restoration of trees along rivers in the crop production landscape to help stabilize soils and improve water quality [51] and to connect patches of suitable habitat [52] to allow wildlife to move through the valley [53] (figure 2). The scenario is based around explorations of IUCN, Kilombero Sugar Company and Reforest Africa looking to restore forests as linear structures of 60 m width either side along riverine areas (as outlined in the Environmental Act 2004; see https://tnrf.org/en/node/9153). Using the *raster* package [54], we mapped the restoration scenario as increase in amount of forest in the landscape, updating the maps of forest cover, distance to forests and percentage of forests within 250 m. We recalculated maps related to canopy closure and its variation in the landscape, using current data on canopy closure variation in forests in the landscape. We updated the covariate raster used in the predictive modelling with the recalculated maps, creating the scenario covariate raster (see §4 for details).

### (b) Collecting data to parameterize models in the framework

We used preliminary field data, with remote sensing data and data from global databases to demonstrate ‘proof of concept’ for stakeholders in FLR interventions targeting our case study and forest-agricultural landscapes in the rural tropics in general. We acknowledge uncertainties from data (unequal sampling of all environmental gradients relating to landscape configuration) and model constraints (no mitigation techniques, no model of animal movement/behaviour changes).

#### (i) Microclimate, crops and biodiversity

In 72 ecological plots of 20 m × 20 m size (forest: 12, woodland: 5, grassland: 7, cropland: 48), representing key crop and non-crop habitats (electronic supplementary material, figure S1), we measured plant health, microclimate and stand structure as leaf fluorescence, leaf surface temperature, ground surface temperature and vegetation canopy closure above 1 m vegetation height following [55]. We collected yield data in a subset of the plots representing important food and cash crops: six okra plots, 18 sugarcane plots and six maize plots.

Our biodiversity data for this study focussed on mammals and birds, both indicators of ecosystem function [48]. Mammals were sampled with 66 camera traps in the study landscape over a total of 4128 camera trap nights. between October 2019 and July 2021 (see electronic supplementary material, figure S1 and S5 for sampling details). Traps were established at different distances from towns and roads and in different land cover types (forest: 33, grassland: 9, cropland 24). In the same season, we implemented bird surveys a minimum of twice by the same expert in 124 points across the landscape (forest: 25, grassland: 10, cropland: 86, village land: 2 and one at the river side).

For each species identified using standard reference guides (see electronic supplementary material, S5), we downloaded the latest IUCN threat information using the *rredlist* package [56] and categorized them as ‘threatened’ if any of the latest threat status was listed as vulnerable, endangered or critically endangered. We assumed that threatened species were of highest biodiversity value. For each species, we extracted information on their diets using the EltonTraits database [57]. Based on the dominant diet item (greater than 50% respectively of the diet), we classified species into those primarily feeding on invertebrates, vertebrates, or plants and seeds (see electronic supplementary material, Dataset D1). We assumed that diet affects the classification of species into those providing services (carnivores controlling pests) or disservices (plant- and seed-eating species).

#### (ii) Crop raiding risks, human wellbeing and other social data

Crop raiding incidents were recorded between January 2019 and June 2020 (see electronic supplementary material, S2 for details) by the Southern Tanzania Elephant Program (https://stzelephants.or.tz/), a local NGO that works to establish community projects that support coexistence between people and elephants. Local enumerators were employed to monitor crop use by elephants, record crop raiding and their spatial location at weekly resolution. Non-crop raiding incidents were generated post hoc from track logs provided by monitors from survey days where no crop damage was recorded.

We used 20 indicators to build a wellbeing composite index based on questions formulated in a household survey, implemented with 461 randomly selected farming households in six villages between October and December 2019 in the study landscape (see electronic supplementary material, S3 for details). These indicators were representative of five wellbeing domains, i.e. ‘basic material for a good life’, ‘health’, ‘social relations’, ‘security’ and ‘freedom of choice and action’, selected using a Wellbeing Indicator Selection Protocol [38]. The indicators used included: financial savings, household wall material, household assets, banking, water access, land ownership, livestock, sickness, health insurance, diet, borrowing of resources, recognition in the village, provision for dependents, provision for self in old age, number of livelihoods, theft security, livelihood satisfaction, nature access, education level and overall quality of life. The variables were normalized, so that all resulting variables ranged from 0 to 1. Each variable was weighted in relation to the number of variables within the corresponding dimension so that all dimensions carried the same weight in the composite index.

We asked additional questions to understand farmers' perceptions of crop damage on their farms due to pests and wildlife. Questions asked included (a) whether or not the survey participant self-reported severe wildlife- or pest-induced crop damage (1: greater than 25% of crop lost, 0: less than 25% of crop lost) within the past 12 months, hereafter referred to as crop damage, and (b) whether or not they self-reported significant damage to crops because of elephants (1: yes, 0: no), and (c) whether participant viewed natural areas as good or bad for their livelihoods (‘4’= very good, ‘3’= somewhat good, ‘2’= neutral/ don't know, ‘1’= somewhat bad, ‘0’= very bad).

### (c) Statistical model underlying pathways in the framework

#### (i) Pathway 1: canopy structure, microclimate regulation and crops

We modelled ground and leaf surface temperature, leaf fluorescence and crop yield as functions of vegetation canopy closure using generalized linear models. Specifically, we tested generalized linear models and generalized additive models, choosing the simpler model when the ANOVA test suggested that models did not differ significantly from each other.

#### (ii) Pathway 2: modelling landscape configuration effects on biodiversity

We used linear discriminant analysis (LDA) to predictively model and map continuous surfaces of biodiversity metrics for birds and mammals in response to environmental attributes characterizing landscape configuration. Our biodiversity indicators included (1) probability of presence (separately for threatened mammals and threatened birds) and (2) probability of observing a low, medium or high number of species of all birds or mammals and of observing a low or high number of species feeding predominantly on plants and seeds (table 1).

**Table 1.** Summary statistics of attributes and indicators in habitat types. Habitat types differed in vegetation attributes, microclimate, biodiversity indicators and crop raiding risk. Woodland and forest had higher canopy closure values (%) compared to grassland and cropland (ANOVA with *post-hoc* Tukey HSD tests: *p* < 0.001). Ground surface temperature (T, ground °C) and leaf surface temperature (T, leaves °C) were lower in forest versus cropland (*p* < 0.05). Leaf fluorescence was higher in forest and woodland versus grassland (*p* < 0.01 each) and in cropland versus grassland (*p* < 0.05). Forest plots had a higher probability for a high number of mammal species (mammals, H) compared to cropland (*p* < 0.01) and a higher probability for observing threatened mammals compared to all other habitats (*p* < 0.01). Forest had a lower probability for presence of threatened birds compared to all other habitat types. Cropland and woodland plots had a higher probability for a higher number of birds (birds, H) versus forest and grassland plots (*p* < 0.05). Grassland had a marginally higher probability for a high number of seed- and plant-eating mammals versus cropland plots (*p* = 0.051). Cropland and woodland had a higher probability for a high number of plant- and seed-eating birds versus grassland and forest (*p* < 0.001). The latter was also higher for grassland versus forest (*p* < 0.01).

	forest	woodland	grassland	cropland
measured on the ground in plots				
*N*	12	5	7	46
*T*, ground (°C)	31.2 ± 3.4	32.1 ± 2.5	33.2 ± 5.1	35.6 ± 4.7
*T*, leaves (°C)	29.7 ± 2.5	30.0 ± 1.6	32.0 ± 2.9	32.3 ± 2.7
fluorescence	40.1 ± 6.3	42.2 ± 5.6	32.0 ± 3.0	38.1 ± 4.6
canopy closure	41.0 ± 16.4	43.5 ± 14.1	8.2 ± 13.9	10.7 ± 12.9
predicted from sensor data and landscape configuration (see S3)				
*biodiversity metrics: probabilities of high number of species (High), of presence of threatened species (Threatened), and of high number of plant and seed eating species (HSP)*				
*N* plots	12	5	7	48
birds, High	0.21 ± 0.12	0.48 ± 0.10	0.21 ± 0.06	0.44 ± 0.17
birds, Threatened	0.05 ± 0.03	0.17 ± 0.06	0.15 ± 0.05	0.17 ± 0.08
birds, HSP	0.02 ± 0.04	0.51 ± 0.06	0.26 ± 0.17	0.52 ± 0.16
mammals, High	0.50 ± 0.36	0.33 ± 0.39	0.39 ± 0.38	0.22 ± 0.27
mammals, Threatened	0.57 ± 0.22	0.26 ± 0.13	0.27 ± 0.18	0.14 ± 0.13
mammals, HSP	0.20 ± 0.24	0.09 ± 0.13	0.28 ± 0.26	0.06 ± 0.19
*human–wildlife conflict metric (HWC): risk of crop raiding*				
HWC	NA	NA	NA	0.41 ± 0.31

We first ran a stepwise forward variable selection on all environmental variables that were not highly intercorrelated (Pearson correlation coefficient less than 0.7) using the *greedy.wilks* function in the *klaR* package [58] based on the Wilk's Lambda criterion. Covariates retained in the stepwise selection were then used as significant predictors in separating classes for each response metric in each final LDA model (see electronic supplementary material, table S6 for details). We used each final model with the baseline covariate raster and the scenario covariate raster to predict and map biodiversity indicators for each landscape pixel. For the example, we predictively mapped the probability of observing a low number of mammal species, medium number of mammal species and high number of mammal species, each for the baseline and for the scenario.

Note that we tested for the presence of spatial autocorrelation effects in each final model. For this, we computed Moran's I for the residuals in the modelled relationships between predictors and the response. The test did not find significant spatial autocorrelation in any of the final models.

#### (iii) Pathway 3: human wildlife conflict and wellbeing

As with Pathway 2, we used LDA to predict the probability of observing a crop-raiding event (*N* = 97 absence points, 308 presence points) from landscape configuration variables. The final LDA model (see electronic supplementary material, S6 for details) was used with the baseline covariate raster and the scenario covariate raster to predict and map the probability of crop raiding for the baseline and for the restoration scenario. This modelling was based on two assumptions: (1) the riparian corridors are not fenced, and (2) elephants will use the riparian corridors in the same way that they currently use forests in the landscape.

We used a radar plot to visualize wellbeing differences between men and women along the five dimensions of wellbeing. We used density plots to visualize wellbeing differences between men and women farmers, distinguishing between farmers who reported on having experienced extensive wildlife-induced crop damage or elephant damage to crops within the past 12 months. We compared these groups for significant differences using ANOVA with *posthoc* Tukey HSD test. We used linear mixed-effects models in the *lme4* package to test for effects of self-reported (household surveys) crop damage (i: through wildlife/pests, ii: through elephants) and gender on wellbeing. We included the village to which the household belonged as random effect, assuming differences between the villages and wellbeing of people within, due to location in the landscape. We assumed that higher crop damage in addition to wellbeing would significantly impact people's positive perceptions of nature. We used the *VGAM* package [59] to implement an ordinal regression model and to test whether farmers and their perception of nature's contribution to their livelihoods were linked to their wellbeing and perceptions of crop damage on their farms, including name of the village as fixed covariates in the model.

### (d) Analysing and visualizing changes in biodiversity and crop raiding

We used lollipop plots to visualize predicted changes in the trade-offs and win–win in biodiversity indicators and crop raiding risk in response to the restoration intervention. We did this at the scale of ecological plots, extracting biodiversity and crop raiding metrics from the baseline and restoration scenario maps. We also did this for the scale of habitat type (mean across plots within habitat type). Our cropland plots may be a biased subset of farmed land in the landscape. We thus used google earth to randomly locate 50 additional sample points in small holder-farmed land and 50 additional points in the commercial sugarcane plantation. We then extracted the baseline and scenario outcomes for those points to subsequently visualize the predicted restoration outcomes for biodiversity and crop-raiding risk for those additional points, at the scale of crop production type (average of each metric per production type).

## 3. Results

### (a) Baselines for biodiversity indicators and crop raiding

Plots differed in their structural and biodiversity attributes (see table 1 for summary statistics). In summary, forest plots had higher canopy closure, lower ground and leaf surface temperature and higher leaf fluorescence compared to croplands. At the scale of habitats, forests were more likely to support a high number of mammals (probability >0.7) and cropland and woodland were more likely to support a high number of bird species (probability >0.4 each). The probability of observing threatened mammals was highest in the forest (>0.7; table 1). The likelihood of observing threatened birds and a high number of plant- and seed-eating birds in forests was very low (<0.2), as was the likelihood of observing a high number of mammal species in croplands or woodlands.

**Figure 3.**
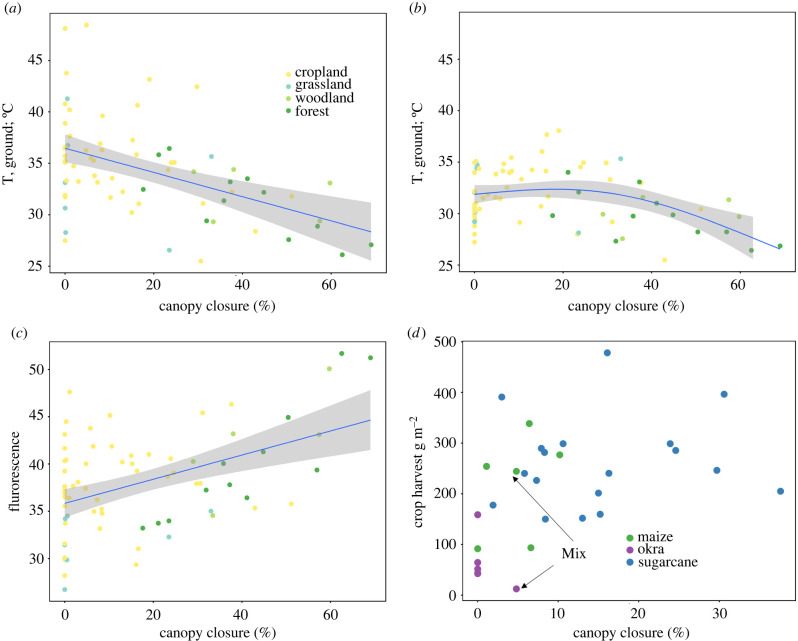
Canopy closure effects on microclimate and vegetation. (*a*) Ground surface temperature declined linearly with canopy closure (general linear model, *N* = 70, degrees of freedom (d.f.) = 68, deviance (*D*) = 22.5, coefficient: −0.117, *p* < 0.001). (*b*) Leaf surface temperature decreased (general additive model, *N* = 71, *D* = 19.4, d.f. = 68, *p* < 0.001) nonlinearly with canopy closure. (*c*) Leaf fluorescence increased linearly with canopy closure (*N* = 71, d.f. = 69, deviance = 26.4, *p* < 0.001). (*d*) Yield in sugarcane (in 0.1 g m^−2^) and okra showed no relationship with canopy closure and maize yield increased slightly (generalized linear model, *N* = 6, d.f. = 4, deviance 15.6%, *p* < 0.01). Mix: maize and okra grown on same plot with yields shown separated by crop type.

Tree canopy closure profoundly modified microclimate but had a weak positive effect on some crop yields only (figure 3). Ground surface temperature decreased linearly with increased canopy closure, with a 1.2°C drop for each ten per cent increased canopy closure (figure 3*a*). Leaf surface temperature decreased nonlinearly with canopy closure, and fluorescence of leaves increased (figure 3*b,c*). Canopy closure was very low for the four okra plots (0–4%) in which we measured yield (figure 3*d*). Maize yield showed a weak linear increase with canopy closure over the observed canopy closure range (0–10%) and sugarcane yield showed no relationship with tree canopy closure (2–34%) (figure 3*d*). Sugarcane can reach more than 3 m height and our canopy closure measurement may in part represent self-shading. Most plots had no trees directly growing within plot boundaries, except for one sugarcane and one maize plot (both with medium range yields), which each feature one tree individual, and one okra plot (the one with the highest yield) with four tree individuals.

### (b) Changes in biodiversity outcomes under the restoration scenario

**Figure 4.**
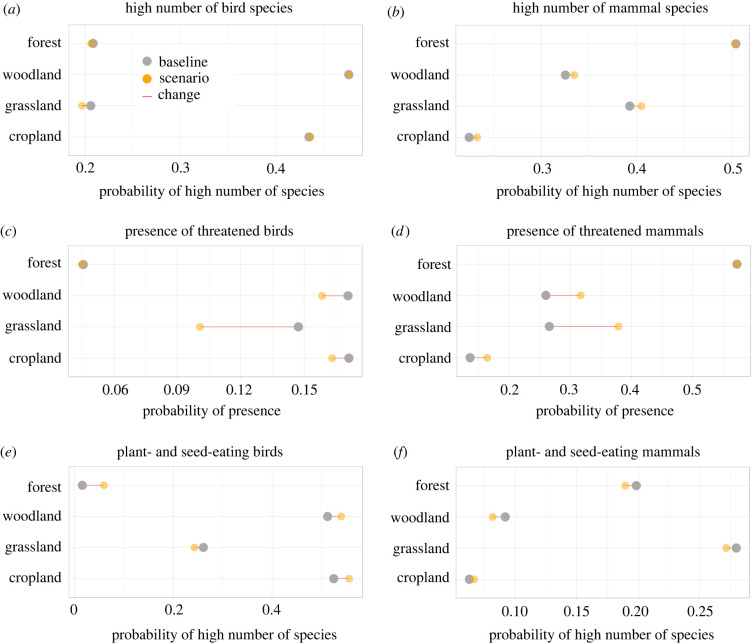
Lollipop plots for biodiversity indicators before and after the restoration scenario. Data averaged across plots at the scale of habitat type (see S6 in the electronic supplementary material for detailed cropland plots data). Grey: baseline outcome. Orange: scenario outcome. (*a*) The probability of observing a high number of bird species declined in grassland from a low starting point. It was higher in woodland and cropland and did not change under the scenario. (*b*) The probability of observing a high number of mammal species increased for grassland by 0.13, for woodland by 0.03 and for cropland by 0.02 and declined for forest by 0.05. It was high in forests before the scenario. (*c*) The probability of observing threatened bird species declined by just 0.05 for grassland and by 0.01 for woodland and cropland, but the likelihood of observing them was low in each habitat type before the scenario. (*d*) The probability of observing threatened mammals increased by 0.10 in grassland, 0.02 for cropland and 0.01 for woodland, and it declined by 0.06 for forest. (*e*) A higher number of plant- and seed-eating birds were more likely to be found in woodland and cropland before the restoration scenario and that likelihood increased for both habitat types. (*f*) A higher number of plant- and seed-eating mammals were more likely to be found in forest and on grassland before the scenario. This biodiversity indicator declined for forests by 0.10 and increased for grassland (mean: 0.06) and woodland (mean: 0.07).

Our models predicted differential effects of the restoration intervention on our biodiversity indicators at habitat scale (figure 4). The probability of observing a higher number of species increased marginally for mammals on cropland, woodland and grassland but declined for birds on grassland (figure 4*a,b*). The probability of observing threatened mammals increased and that of threatened birds declined in all habitat types but forests, where it remained constant (figure 4*c,d*). or a high number of mammal species generally increased under the restoration scenario except for within forests (figure 4*b,d*). The probability of observing a higher number of plant- and seed-eating bird species increased slightly for all habitat types but grassland (figure 4*e*). Under the restoration scenario, the probability of observing a higher number of plant- and seed-eating mammals was predicted to decline in all but cropland, for which it increased marginally (figure 4*f*).

Within croplands, we found high variability in biodiversity outcomes under the restoration scenario (electronic supplementary material, figure S7). The baseline probability of observing threatened species was low across cropland plots, exceeding 0.5 in two plots for mammals and 0.4 in two plots for birds. Under the restoration scenario, the probability of observing threatened mammals was predicted to increase in six plots (mean: 0.22) and for birds it was predicted to decline weakly in eight (mean: −0.05). Predicted increases in the probabilities of observing threatened mammals and predicted declines for those of birds were robust across crop production systems under the restoration scenario (electronic supplementary material, figure s8).

The baseline probability of observing a high number of mammal species on cropland was low, exceeding 0.5 in seven of the 48 cropland plots. Under the restoration scenario, that probability was predicted to decline marginally in nine plots (mean: −0.01) and increase marginally in eight (mean: 0.06). Across crop production systems, this probability increased (see electronic supplementary material, S8). The baseline probability of observing a high number of bird species on cropland exceeded 0.5 in 50% of the cropland plots and the restoration scenario was predicted to decrease this probability in three plots (mean: −0.02) and increase it in 5 (mean: 0.02). However, patterns differed between crop production systems, as probabilities of observing high numbers of bird species were predicted to decline in the randomly chosen plots representing small-holder and industry farming approaches (see electronic supplementary material, S8).

The baseline probability of encountering a high number of plant- and seed-eating bird species on cropland exceed 0.5 in 33 plots (and 0.6 in 19). Under the restoration scenario, this probability was predicted to increase in 31 of the plots (mean: 0.09) and decline in 5 (mean: −0.23). At the scale of crop production systems, smallholder farms were predicted to experience a decline, no discernible change was predicted for the industry farm and an increase was predicted for our ecological plots. By contrast, the baseline probability of encountering a high number of plant- and seed-eating mammal species was below 0.1 in all but four plots. Under the restoration scenario, this probability was predicted to increase in 4 plots (mean: 0.09) but decline in 9 (mean: −0.01). All crop production systems were predicted to see increased probabilities of observing a high number of plant- and seed-eating mammals, but starting from very low probabilities either way.

The baseline probability of observing carnivorous species on cropland was very high for mammals, exceeding 0.6 for 38 plots, and varied mostly between 0.2 and 0.4 for birds. Under the restoration scenario, the probability of observing carnivorous mammals declined in five (mean: −0.07) and increased in 29 (mean: 0.03) plots; and the probability of observing carnivorous birds was predicted to decline in five plots (mean: −0.21) and increase weakly in 17 (mean: 0.04).

### (c) Changes in crop raiding risk under the restoration scenario

**Figure 5.**
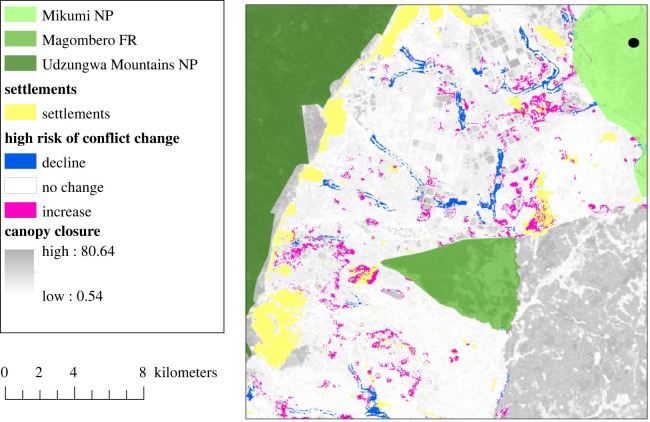
Prediction of change in conflict outcomes under the restoration scenario. The restoration intervention focussed on restoration of trees along rivers and creeks. The modelling was based on two assumptions: no mitigation/prevention techniques exist around the restored habitat and wildlife will use the restored forests in the same way that they currently use other forest in the landscape. The crop conflict map shows the change in high risk (*p* > 0.5) areas before and after the restoration and predicted a spatially localized increase in crop raiding risks in parts of the landscape. FR, forest reserve; NP, national park.

Our model predicted changes in the spatial distribution of high crop raiding risk in cropland areas under the hypothetical restoration scenario considered here (figure 5). This predicted pattern was robust across crop production systems (see electronic supplementary material, S8). For our ecological plots, crop raiding incidents were predicted to become more likely in 27 plots under the restoration scenario (mean = 0.07), while three showed declines in the probability of conflict (mean = −0.10) (electronic supplementary material, figure S7). The risk of crop raiding increased from a mean of 0.51 to 0.54 on smallholder farms under the restoration scenario.

### (d) Crop damage, human wellbeing and perceptions of nature

While men and women had similar perceptions along the five dimensions of wellbeing included in our analyses (electronic supplementary material, figure S9), wellbeing was slightly higher for men versus women farmers interviewed (ANOVA, *p* < 0.001). While gender was significant in predicting wellbeing of farmers (AIC −363 compared to AIC −351 for null model with village as random effect), self-reported crop damage in general or self-reported crop damage through elephants were not.

**Figure 6.**
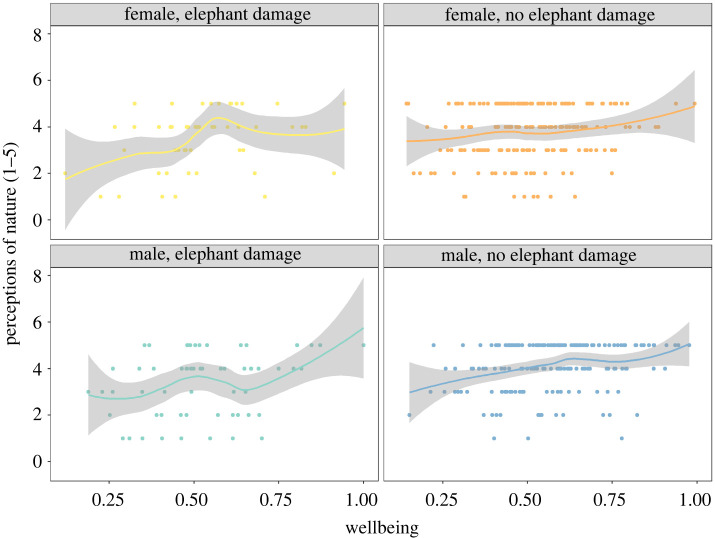
Community perceptions of nature's value (ordinal scale). Data were collected in household surveys. People's perceptions of nature as being of ‘high value’ increased with household wellbeing (ordinal regression model, AIC 1297, degrees of freedom 1839), with significant effects of the gender of the survey participant but no effect of self-reported crop damage through elephants.

People's perceptions of nature as being of ‘high value’ increased with household wellbeing (figure 6). The ordinal regression model explaining this relationship (AIC 1297, degrees of freedom 1839) performed better than the baseline model (AIC = 1326, degrees of freedom 1840, ANOVA *p* < 0.001). The best fitting model explaining people's perception of nature included household wellbeing, gender of survey participant and village name (AIC = 1258, degrees of freedom 1832), but not self-reported crop damage through either wildlife and pests or elephants. In brief, if farmers had lower wellbeing (model coefficient: 2.43, *p* < 0.001) and were women (model coefficient: −0.51, *p* < 0.01), they were less likely to give a high score for their perception of nature's contribution to their livelihoods. Two of the villages had a negative coefficient (Msolwa and Msalise), while the others had a positive coefficient (Kidatu, Mangula, Mgudeni and Sanje).

## 4. Discussion

We demonstrate for the first time how a new systems approach framework can be used with a new protocol for wellbeing assessment to evaluate and map likely changes in ecosystem services and disservices following tree restoration in a crop production landscape of high biodiversity value. The data used to parameterize the framework are preliminary, and results have to be treated cautiously. Yet, early findings from this ‘proof of concept’ study suggest—at landscape scale—likely larger benefits of tree restoration interventions for mammal biodiversity indicators, likely smaller losses for bird diversity indicators, and a potential increase in the risk of crop raiding (assuming no mitigation measures are implemented). These changes in response to restoration are spatially variable and directly linked to changes in landscape configuration. Crop loss to wildlife incurs local costs to people's livelihoods [60], which largely depend on income from crops. Yet, impacts on the wellbeing of people across the five wellbeing dimensions are currently negligible and do not affect people's perception on the value of nature for people's livelihoods. This may suggest that tree restoration interventions, if managed carefully using spatially targeted mitigation measures, can have positive net outcomes at landscape scales.

### (a) Pathway 1: canopy structure, microclimate regulation and crops

Our data support findings from forest degradation landscapes, globally, which suggest that tree restoration likely has benefits for climate change adaptation [61,62]. In our study, higher tree density and thus canopy closure on farms reduce surface temperatures of vegetation on the ground, with positive effects on fluorescence of ground vegetation and few to no impacts on crop yields. Our data demonstrate that even when averaged at the scale of plots, surface temperatures of leaves and ground in our study landscape can easily exceed 35°C and may at times exceed 50°C (measured as surface temperatures and using air temperature data, M Pfeifer 2021, unpublished data). The actual effects on crops produced in our landscape are not yet determined. But evidence from field trials on major world food crops and vegetation models [63] is robust and shows that while agricultural crops vary in their responses to changes in temperature, rainfall and carbon dioxide, heat stress produces consistently negative impacts. Expected warming and drying in Africa have been suggested to reduce crop yields by 10–20% by 2050 and simulations for maize and bean yields suggest that losses may even exceed 20% in the Kilombero Valley [64]. Heat stress acts to reduce leaf photosynthesis and decreasing grain number on cereal crops [65], and even short heat waves can significantly reduce crop yields [63]. Irrigation and shifts to shorter production cycles may be able to address yield decline risks from shifting temperatures [66], but require costly infrastructure investment. Our data show that tree canopies can introduce shade and thereby buffer crops and other vegetation on the ground from rising temperatures, whether these are short-term episodes imposing heat stress or long-term and continuous. In this study, we did not explore the restoration of trees on our farm plots. However, the models used in the systems approach could be used to predict the outcomes of that specific scenario. We stress that crop and soil management practices can further modify the relationship between temperature, stress and crop yields [67]. And restoring riparian corridors can improve water quality, reduce flood risk and soil erosion and regulate permanence of water supplies, which are important services in crop production landscapes. There are additional pathways in our framework (figure 2) that link land configuration and management to outcomes, and these will be included in future updates to our predictive modelling and mapping.

#### (i) Restoration relevance

The microclimate buffering effect provided by tree canopies is well established in the context of forest degradation [68,69] and forest fragmentation [55]. In the context of smallholder farming, trees on the farm have been suggested to provide an insurance against risks from heat stress, and anecdotally have also been shown to reduce risk from insect pest outbreaks and leaf herbivory in shaded cacao agroforestry [10]. Smallholder farmers are seen as highly vulnerable to climate variability and change, with limited financial capacity to adapt, a lack of safety nets and high reliance on natural resources [70]. Our findings lend support to promotion of tree restoration in crop production landscapes to increase climate change resilience of farmers in the face of projected climate change. However, the choice of trees needs to be considered carefully against criteria of species origin (native/invasive), species adaptation to local conditions (and resilience to climate change) and species traits linked to services (production of biopesticides, contribution to soil fertility) and disservices (crown shade and root growth and their interaction with crop growth). The discussions of benefits and costs using data have potential to be influential for engagement with farmers who may already have an interest in tree planting and with farmers who farm nearby areas that are being restored, promoting local involvement in the restoration planning and helping to mitigate expected trade-offs because of planned interventions.

### (b) Pathway 2: landscape configuration and ecological communities

Birds and mammals that can be found in tropical forest—agricultural landscapes differ in their habitat needs and dependencies, in our case study landscape and elsewhere [71]. Our data suggest that croplands, and in particular smallholder farms that practise agroforestry, can support a high number of bird species and in particular plant- and seed-eating birds. This aligns with findings from a similar study landscape in Kenya, which also comprised natural humid forests neighboured by an industrial sugarcane plantation and smallholder farms that integrate trees and other natural habitats with crops [72]. Our landscape provides a diverse mosaic of habitat patches—in particular, on land farmed by smallholders—and tree patches can be found scattered throughout, potentially allowing many forest-dependent bird species to move through the valley and between larger forest habitats. By contrast, we found that the farmed land in principle was less supportive than forest or grassland for mammal diversity, except for carnivorous species. Our predictions suggest that the planned restoration intervention may—on average and at landscape scale—benefit mammal diversity in cropland, grassland and woodland habitats and contribute to increases in the numbers of plant- and seed-eating birds. However, predictions also suggest declines in other bird diversity indicators, including of threatened birds.

We emphasize caution when interpreting our findings. First, we focused on a subset of indicators for biodiversity. While we distinguished between species that may provide services (carnivores controlling pests) or disservices (plant and seed-eating species), follow-up analyses (e.g. exclusion experiments) would be needed as well as consideration of other taxa and in particular insects and plants. Insects as a key taxonomic group for crops, acting as pollinators, pests or pest controls and individuals collected from plots, are currently being identified. Second, current data constraints may reduce model accuracy. We did not correct for detectability when estimating bird species richness for each survey point, although any uncertainty introduced may be small as we carefully controlled for covariates of detection probability before data collection [73]. Each survey point was surveyed multiple times (2–4) on different days, at different times (early morning and late afternoon) by different surveyors who have extensive expertise in bird surveys across Southern and Eastern Africa. A subset of measurements confirmed findings against a second observer operating across the same points.

#### (i) Restoration relevance

Applying the framework allowed us to map biodiversity outcomes in the study landscape predictively before any tree had been planted. These predictions, while currently uncertain, allow us to see which areas are likely to benefit from the tree restoration in the landscape in terms of biodiversity values, but also which areas may experience some declines. Furthermore, we can consider how specific outcomes are likely to change depending on ‘how much area’ and ‘in what configuration’ trees are being restored in a landscape using FLR. Riparian buffers, for example, are suggested to differ in their effectiveness in delivering on ecological outcomes because of differences in vegetation type (e.g. trees are more effective at removing pesticides than shrubs [74]), quality and buffer width. Evidence from Costa Rica and India suggests that it is possible to sustain relatively high biodiversity in crop production landscapes if these are heterogeneous and incorporate elements of naturally occurring habitats [71]*.* Additional pathways that could be explored are the planting of trees that carry multiple functions, benefitting soil or wildlife or both, analysing how this may affect trade-offs between ecological and wellbeing outcomes. Yet, evaluations need to be designed carefully and explored with stakeholders using maps (e.g. where planting trees increases pest control and where it attracts plant- and seed-eating birds, damaging crops) to capture benefits and costs and instigate spatially targeted mitigation measures if needed.

### (c) Pathway 3: landscape configuration, human–wildlife conflict and wellbeing

Our models estimate that the baseline risk for elephant crop raiding is spatially highly variable in the landscape but can exceed 70% for several smallholder farms. Our models predict that changing landscape configuration under the restoration scenario may increase crop raiding risk in some areas and decrease it in others (figure 5). We highlight that crop raiding incidents have not been sampled equally across the entire landscape and we have not yet accounted for other drivers of crop use by elephants, including rainfall seasonality, availability of wild food plants and crop production cycles [75]. In the models, we did not account for hard fencing or other elephant deterrent methods (e.g. beehive fences and olfactory repellents), which may be used to accompany the restoration of linear forest structures and may reduce the probability of elephants moving into adjacent farmland to raid crops. Also, elephants may use narrow forest corridors traversing human-inhabited land differently compared to larger forest refuges, making faster and more directional movements [76,77]. Evidence of relationships between risk of crop raiding and distance to corridors is inconclusive [78,79]. In the sister project CORRESTOR, we will close data gaps and improve predictive models based on mitigation techniques trialled in the landscape and exploring the use of individual-based models to capture animal movement.

Our household data suggest that the impacts of current levels of self-reported crop damage on the wellbeing of smallholder farmers seem to be negligible. We also did not find an expected association between food security or household income and self-reported crop damage in our household data. However, farmers may over- or underestimate crop damage incurred to them by wildlife. And, we did not measure wellbeing in the few communities located in identified elephant–crop conflict hotspots (e.g. Magombera). We suggest that self-reporting of conflicts may be uncertain and deserves further explorative analyses, using wider-reaching surveys and targeted knowledge-exchange workshops. The degree of vulnerability to negative human–wildlife interactions can differ among individuals because of age, gender, ethnicity, farm location, cultural rules and crop assemblages [80]. Men appear to perceive crop damage more negatively than women, but patterns are weak. From our workshops we found that men preferentially tend to focus on cash crops and women on subsistence crops (i.e. gender-defined livelihood activities, roles and responsibilities), which is representative of systems elsewhere in Tanzania.

The literature suggests that farmers' wealth and their subsequent ability to access land, labour and capital [81] may affect their resilience to crop damage risk and tolerance to conflict. A study from South Africa also found higher farmer tolerance for primates and ungulates, but lower tolerance for carnivores and birds [82]. In our landscape, perceptions of nature's value are high for people who have higher wellbeing, whether crop damage was high or not, but this perception was lower for women. How perceptions of nature's value and wellbeing will respond to changed risks will require careful monitoring following actual forest restoration, taking into account gender dimensions, location and livelihoods in the monitoring design.

#### (i) Restoration relevance

Systematic consideration of the costs and benefits of ecosystems experienced by stakeholders in a landscape targeted by restoration is required to support restoration planning and anticipate outcomes of potential interventions [83]. Individual stakeholders will have different experiences of these costs and benefits of ecosystems at different spatial and temporal scales. Our method framework allows us to identify and map at least some of these anticipated trade-offs, delineating regions within the landscape that require targeted mitigation and management to minimize costs. Human–wildlife conflict and its spill-over from protected areas have been challenges for conservation [60,84]. Appropriate conflict prevention measures can play a critical role for farmer livelihoods. Tolerance increases when the benefits of wildlife are established, while displacement from land and damage to livelihoods can create negative attitudes toward living with wildlife [85]. Restoration planning will have to dedicate sufficient resources and capacity training, working with farmers and other stakeholders, to identify and evaluate tools that can be effective in managing human–wildlife conflicts and monitor their effectiveness as the dynamics of the socio-ecological system are changing [86]. The next step requires us to test whether and to what extent information generated from our models and maps using the systems approach framework can be used in participatory knowledge exchange workshops with different stakeholders. This step is crucial to (i) ground model results in local realities and identify stakeholders who are more negatively impacted by expected trade-offs, and (ii) explore barriers and challenges to implementing possible solutions that sit within the contexts, values and knowledge systems in which the communities operate.

#### (ii) Upscaling potential

Examining the transferability of the systems approach for identification of restoration opportunities in other regions is critical. Remotely sensed data may prove useful in this context, allowing scaling in space and time, strengthening the capacity of the systems approach framework to capture the complexity of driver–outcomes relationships [19,20]. In doing so, we may gain understanding of which variables are repeatedly important in calibrating the model pathways, and thus may be the most important to focus on with ground data collection efforts. Through integration of a wider variety of sensors (hyperspectral, LiDAR, RADAR) and platform types (UAV, aircraft, satellite) and other global datasets (WorldClim (http://www.worldclim.com/version2), GBIF (https://www.gbif.org/)), various ecological factors, including those used in this study, could be derived across multiple regions. Social data are highly context-dependent, yet there may be potential in utilizing non-traditional data streams as proxies for traditional survey data to allow wellbeing to be assessed in new areas and at larger scales and combined with localized data sets. Survey data, such as those used in this study, could be collected and analysed to: (1) identify factors relevant to wellbeing; and (2) produce wellness scores or ‘archetypes’ for each chosen analysis unit (e.g. village or farm). These factors could then be extracted from data streams potentially including anonymized Call Detail Records, Mobile Financial Services, and features extracted from remotely sensed datasets (village or farm area, night-time light, road and building condition and distances to nearest larger settlement or water source). Whether the use of remote sensing data would potentially enable the identification of restoration opportunities on a global level and unbiased assessment of sites with the greatest restoration need remains to be tested.

## 5. Conclusion and next steps

The systems approach framework for evaluating tree restoration in forest-agricultural landscapes provides a robust structure for identifying data collection needs to monitor local interpretations of ecological and human wellbeing outcomes. The spatially explicit nature of the approach and its system lens provide significant advancement to previous tools. The model can be as complex as required (interaction of species, networks, governance and management rules), but it needs to have indicators in place that can capture the pathways. Applying the approach, calibrating and validating model pathways to the case study analysed allows us to predict likely consequences of tree restoration before any trees are planted. It can delineate—in space—benefits and costs that are likely incurred to local communities, and in particular to farmers, who comprise an important and diverse stakeholder group in the rural crop production landscapes of the tropics. These may be farmers who already are experiencing crop raiding or who may be located near hotspots of conflicts and farmers who are more likely to be impacted by the restoration process. While current levels of self-reported crop damage through wildlife do not directly affect the aggregated human wellbeing index, how increased risks or new risks in previously unaffected areas following the restoration would translate into changes in wellbeing would need to be assessed. While this could be implemented following the restoration intervention, this would create the ethical dilemma of experimenting with people and their livelihoods. We instead suggest that our findings should be discussed in workshops with farmers to identify their perceptions of possible scenarios of change. The maps produced by the modelling can be used in participatory discussions with these stakeholders to identify landscape- and context-specific solutions that can positively transform the landscapes and lives of people within the environmental, socio-economic and governance constraints and the opportunities that they face. These maps can also form the base from which to identify opportunities for knowledge exchange and capacity building on mitigation measures. In our case study, this means we can, pending future model updates, use the outcomes of the modelling to guide the restoration planning, implementation, monitoring and evaluation as a participatory, evidence-led process. This process can for example include the exploration of the restoration potential of agroforestry and other agroecological practices, a tool for sustainably intensifying crop production [87], building on agronomy and traditional knowledge [88]. The systems approach framework thus allows us to take a proactive approach to restoration trade-offs rather than a reactive one.

Restoration planning has struggled to improve on forest governance and land tenure challenges, considered important for effective and equitable restoration [89], and has given insufficient attention to key threats that play out in complex pathways to produce degraded and deforested land [90,91]. These challenges can be addressed by getting a better understanding of the commonalities and differences in modelled relationships characterizing the framework's pathways for forest-agricultural landscapes that differ in governance and land management contexts. Hence, the next step is to transfer the approach to crop production landscapes elsewhere, collecting key data required to parameterize the modelled pathways and analysing and interpreting their response to restoration scenarios with stakeholders. Ideally, this will also allow us to define those variables that repeatedly come up as important in defining the social and ecological processes and their interactions.

## Data Availability

The data S1–S9 can be found online in the electronic supplementary material [92].
